# The goldilocks effect: the rhythms and pace of hospital life

**DOI:** 10.1186/s12913-018-3350-0

**Published:** 2018-07-06

**Authors:** Jeffrey Braithwaite, Louise A. Ellis, Kate Churruca, Janet C. Long

**Affiliations:** Centre for Healthcare Resilience and Implementation Science, Australian Institute of Health Innovation, Macquarie University, Level 6, 75 Talavera Road, North Ryde, NSW 2109 Australia

**Keywords:** Pace of life, Busy-ness, Staff satisfaction, Patient outcomes, Efficiency, Optimal hospital performance, Complexity

## Abstract

**Background:**

While we have made gains in understanding cultures in hospitals and their effects on outcomes of care, little work has investigated how the pace of work in hospitals is associated with staff satisfaction and patient outcomes. In an era of efficiency, as speed accelerates, this requires examination.

**Discussion:**

Older studies of pace in cities found that faster lifestyles were linked to increased coronary heart disease and smoking rates, yet better subjective well-being. In this debate we propose the Goldilocks hypothesis: acute care workplaces operating at slow speeds are associated with factors such as increased wait lists, poor performance and costly care; those that are too fast risk staff exhaustion, burnout, missed care and patient dissatisfaction. We hypothesise that hospitals are best positioned by being in the Goldilocks zone, the sweet spot of optimal pace.

**Conclusion:**

Testing this hypothesis requires a careful study of hospitals, comparing their pace in wards and departments with measures of performance and patient outcomes.

## Background

Hospitals are fast-moving workplaces characterized by the brisk walk of staff in corridors, quick turnover of patients in wards and the rapid response of emergency teams. We notice no one has studied this aspect of hospital culture.

In the 1970s, psychology researchers began observing the speed at which pedestrians walk and carry out everyday tasks, uncovering significant differences in the rapidity of life between cities and countries [1, 2]. Arguing that such momentum plays a central role in defining the personality of a setting, Levine and Norenzayan showed how pace of life is intertwined with the social-psychological and community characteristics of the cities they observed [3]. In this study, Bern, Zurich, Dublin and Frankfurt were fastest; New York, Singapore and Toronto were next; and Rio de Janeiro, Jakarta and Mexico City were slowest. Later, Levine proposed that the level of “busy-ness—the summation of both speed and activity” [4] was a window into the inhabitants’ culture.

Much like cities, every organisation has a uniquely patterned culture. A good culture, one that is productive, collegiate and supportive, is related to positive patient and organisational outcomes [5]. But busy-ness and the pace of hospital organisational life have not been measured (see Table 1, Glossary of terms). We propose that the tempo of staff is an important variable in hospital culture, and ultimately, in performance, that can be objectively measured.

**Table 1:** Glossary of terms

Concept	Definition
Busy-ness (of hospital organisation life)	For Levine (2005), busy-ness was a state of or subjective experience of having a lot to do, and perceiving that you don’t have much time for other activities beyond your current work, with two components: speed and activity. When you are busy, you perceive that you are not available to complete much else, and that you need to work or concentrate on a task or tasks.
Pace (of hospital organisation life)	The rate of at which clinical and non-clinical work is being progressed; the psychological or subjective experience of rapidity of work, and density of perceptions of this.
Speed of hospitals (also referred to as the tempo of hospitals)	The relative rapidity of staff movement, and rate of activities being done e.g., walking pace between wards, time available to do a drug round; or as a global measure of staff activities.
Briskness	Staff quickness; their liveliness and vigour.
Step up or stepping up the pace	The act of increasing speed or pace: a feeling of accelerating busy-ness.
Four-hour rule in Emergency Departments	Based on the UK experience, Australia introduced the National Emergency Access Target (NEAT) in 2011. This states that patients should be admitted, discharged or transferred within 4 h of arrival, in Emergency Departments (Ngo, H. et al. 2018; Sullivan, C. et al. 2014).
ETTO principle (efficiency-thoroughness trade-off)	This principle, expounded by Hollnagel (2009), suggests that people must make a trade-off between the resources of preparing an activity and the resources in completing that activity. This could be a trade-off of thoroughness over efficiency, or efficiency over thoroughness. Thoroughness and efficiency cannot be maximised at the same time.

### Factors contributing to the unique personality of a hospital

Many factors influencing the speed of hospitals are generic. These factors are mostly linked to the context and nature of the tasks being performed, and the modern drive for efficiency [3, 6–8]. Different units have different activity patterns, however [9, 10]. In Emergency Departments, for example, the pace is two-speed: in downtime, and when there is a rush of patients—a kind of choreographed efficiency. On the more predictable rehabilitation wards, the daily routine is more or less measured and orderly. In palliative care, on the other hand, there is a slower, gentler, and more respectful pace [11]. Theatres have a distinguishable tempo: anesthetized patients lie motionless while surgeons hover above them, then all is speed and activity as drapes and gowns are pulled off, doors thrown open and trolleys reshuffled, ready for the next procedure.

But, like the research on cities, there are grounds for believing that each hospital has a distinctive personality, reflecting “what is valued, the dominant managerial and leadership styles, the language and symbols, the procedures and routines, and the definitions of success that make a hospital unique” [12]. Levine and Norenzayan, for example, found that economically more productive cities in cooler climates, with more individualistic cultures, and higher levels of well-being, had a brisker pace of life. Perhaps, consistency in pace in hospitals might reflect not only such facets as the stability of the culture, but also the professionalism of the clinical workforce, and financial performance of the organisation.

### Increasing pressures on hospitals to step up

Is a faster speed, representing productivity and performance, always a good thing? Levine and Norenzayan indicated that the inhabitants of briskly-paced cities were economically better off and enjoyed subjectively better levels of well-being, but had higher smoking rates, and were associated with increased morbidity due to coronary heart disease [3]. Faced with ever-increasing cost constraints as well as demands for services, hospitals have experienced accelerating pressures to step up the pace [6, 13]. For example, time-based targets, such as the rule in Emergency Departments in the United Kingdom and Australia that people need to be seen within four hours [14, 15] and throughput pressures exemplified by elective surgery wait lists [16, 17] are pushing staff to fit more and more tasks into a set amount of time. Work is expanding in other ways too, such as with the introduction of burdensome documentation, rules and regulations [18]. By stepping up the pace, hospitals may be compromising their delivery of high quality patient care [19]. Indeed, we know that patient satisfaction is lower when nurses and patients feel too rushed [20], while quality of care has been linked to how much time hospital staff spend with patients [20–22]. In the complexity of performance, Hollnagel refers to this as the ETTO—the Efficiency-Thoroughness Trade-Off [23].

Exacerbating this, burnout rates among doctors are reportedly rising, and reaching epidemic levels [24]. Stress and fatigue highlight the problem of hospitals with too few resources and too many demands, which may cause staff to extend their pace beyond that which is sustainable.

**Fig. 1:**
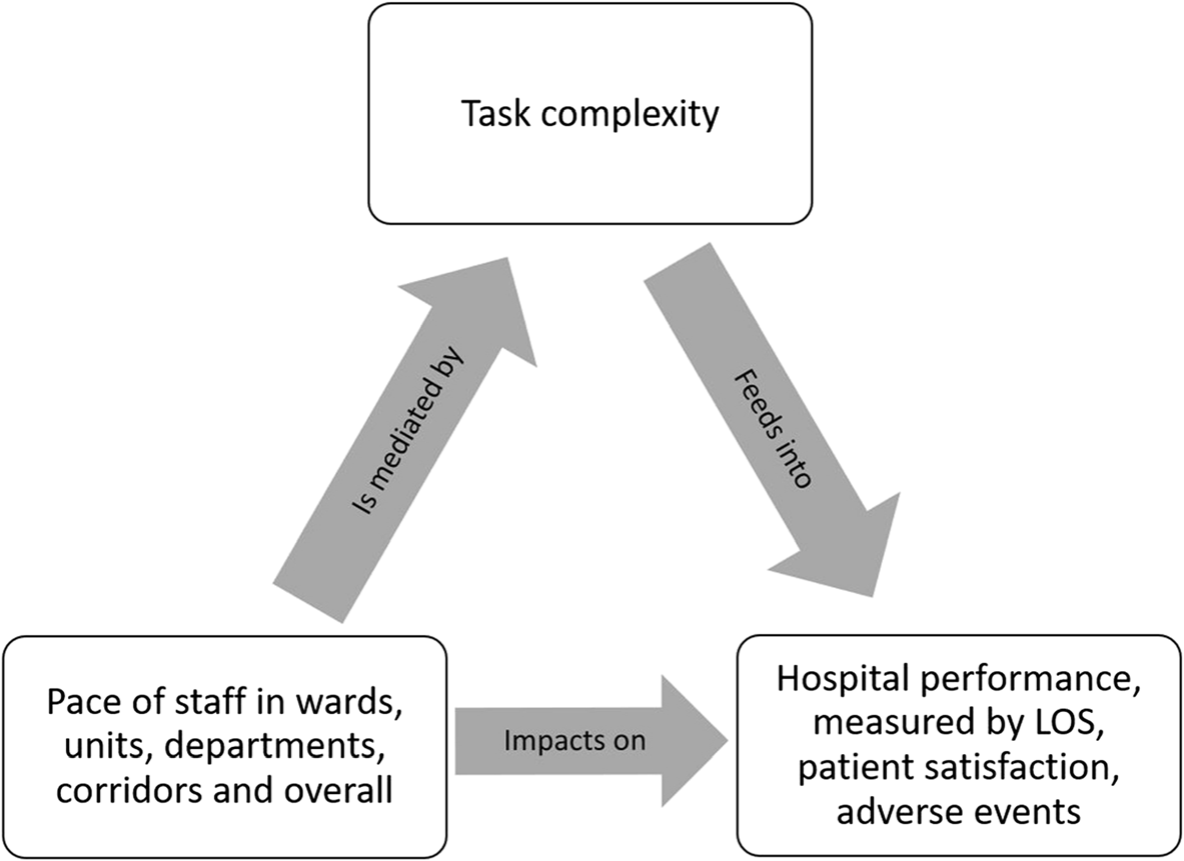
Underlying theoretical model

**Fig. 2:**
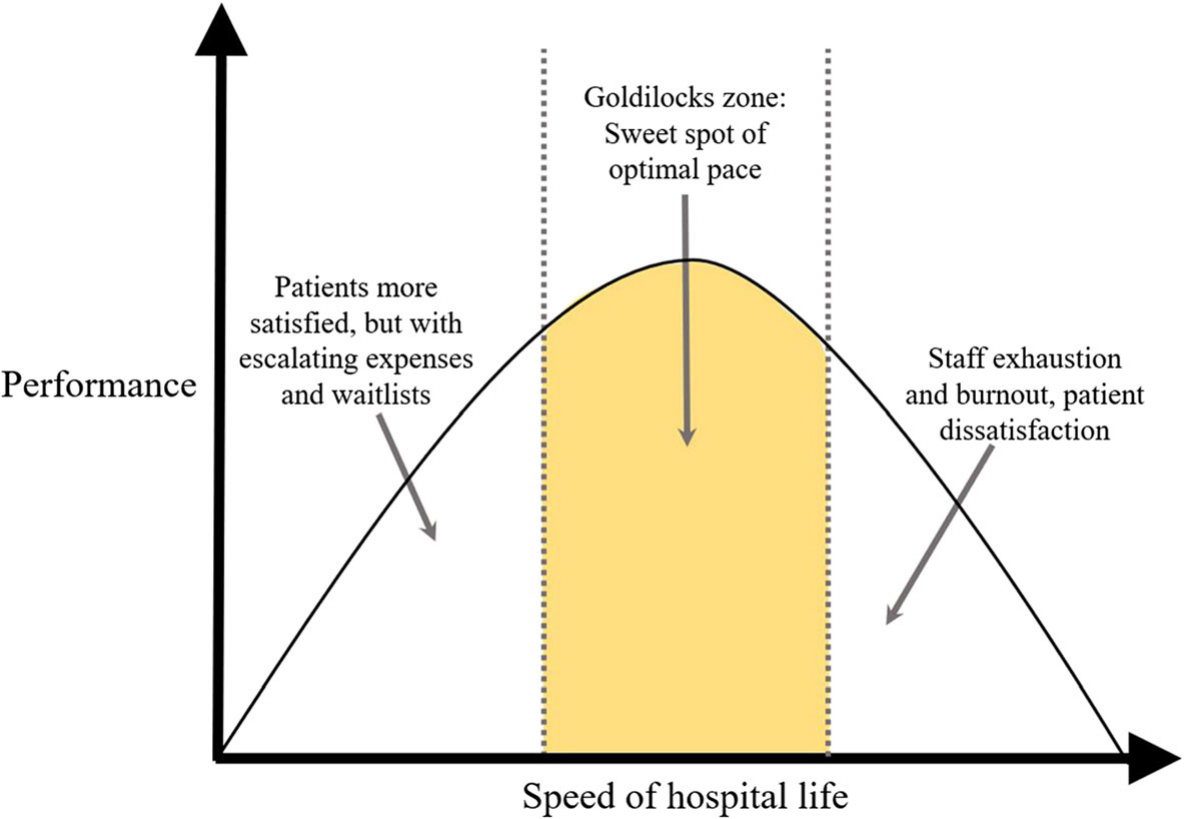
The pace normal distribution curve

Perhaps there is an *optimal* pace for hospitals to function well: too fast and mistakes are made, staff leave exhausted and burnt out, and patients are dissatisfied with their care; but too slow and things do not get done at day’s end, boredom settles in, expenses escalate, and wait lists blow out. Thus, the underlying theory is that pace is related to performance, as depicted in Fig. 1. Based on the Yerkes-Dodson law of the relationship between arousal and performance [25], we suggest that the cultural sweet spot is the Goldilocks zone of the inverted-U function of briskness, where external demand and internal motivations, and the tension between efficiency and quality, are in equilibrium (Fig. 2).

In recognition of the concavity of the curve, we can therefore model performance in hospitals as a quadratic function of the pace.

$$ \sigma =\mathrm{f}\left(\mathrm{P}\right)={\mathrm{aP}}^2+\mathrm{bP}+\mathrm{C} $$

where: σ = performance; P = pace, and a, b, c = coefficients to be determined.

We assume that:

- The pace of hospital life is related to hospital performance, measured by length of stay, and other efficacy measurements.
- When pace is at its minimum or maximum, performance is at minimum.
- Performance is best under conditions of intermediate pace

**Fig. 3:**
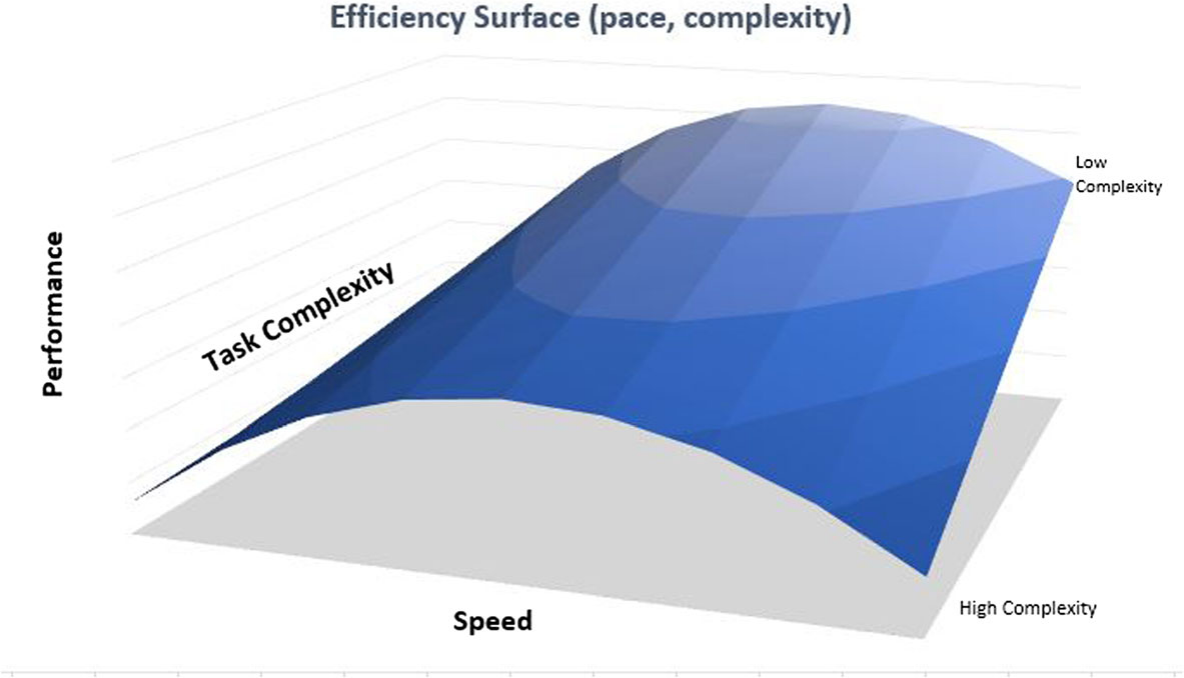
3D surface plot of performance, complexity and speed

Supporting our hypothesis, research applying the Yerkes-Dodson law found that different tasks require different levels of arousal for optimal performance [25, 26]. Specifically, the law holds that easier tasks require higher levels of arousal (e.g., faster pace) for optimal performance than more difficult tasks. So in accordance with such previous research, we propose that the optimum level of pace is likely to be lower for more complex, unfamiliar or difficult tasks, and higher for simple or familiar tasks requiring endurance and persistence. This can be graphed as a 3-D performance surface (Fig. 3).

### Getting to the goldilocks zone

Despite hospital tempo receiving no substantial attention to date, there is much to study here. Differences in the cultural characteristics of workplaces may be expressed in different paces of hospital life and distinctive organisational personalities. For example, the particular way that nurses routinely communicate with each other at change of shift—structured, comprehensive and open, or ad hoc, shallow and defensive—determines at what speed handover of patients should proceed [9]. Tempo can also reflect the local organisational pecking order, in which the most powerful player sets the agenda and the less powerful must accommodate their speed to suit (e.g., theatre staff made to wait for a tardy surgeon). There may, too, be mismatches in cultural expectations (“I’m on time, why aren’t you?”) [27].

If speed is something perpetuated and normalised in the culture of an organisation through the staff routines [9], as well as reflecting something about the organisation’s collective personality and context, then this suggests that through conscious and concerted effort, pace is amenable to change. So, we submit that efforts to decrease a pace that has become uncomfortably or dangerously fast, or stepping up the tempo of an inefficiently slow one, should spring from a collective recognition that speed can affect quality of care, staff well-being, and hospital performance.

## Conclusion

Just like Goldilocks’ porridge, not too hot, not too cold, but just right seems to be the sweet spot. This is a hypothesis worth postulating. We’ll only know if we put the argument to an empirical test. That will involve a carefully designed study of multiple hospitals, comparing their rhythm and pace in wards, corridors, emergency departments and specialised units, and hospital-wide, with measures of their performance. That’s the next step.
